# Anti-adaptors use distinct modes of binding to inhibit the RssB-dependent turnover of RpoS (σ^S^) by ClpXP

**DOI:** 10.3389/fmolb.2015.00015

**Published:** 2015-04-23

**Authors:** Dimce Micevski, Jessica E. Zammit, Kaye N. Truscott, David A. Dougan

**Affiliations:** Department of Biochemistry, La Trobe Institute for Molecular Science, La Trobe UniversityMelbourne, VIC, Australia

**Keywords:** anti-adaptor, regulation, general stress response, AAA+ protease, degradation

## Abstract

In *Escherichia coli*, σ^S^ is the master regulator of the general stress response. The level of σ^S^ changes in response to multiple stress conditions and it is regulated at many levels including protein turnover. In the absence of stress, σ^S^ is rapidly degraded by the AAA+ protease, ClpXP in a regulated manner that depends on the adaptor protein RssB. This two-component response regulator mediates the recognition of σ^S^ and its delivery to ClpXP. The turnover of σ^S^ however, can be inhibited in a stress specific manner, by one of three anti-adaptor proteins. Each anti-adaptor binds to RssB and inhibits its activity, but how this is achieved is not fully understood at a molecular level. Here, we describe details of the interaction between each anti-adaptor and RssB that leads to the stabilization of σ^S^. By defining the domains of RssB using partial proteolysis we demonstrate that each anti-adaptor uses a distinct mode of binding to inhibit RssB activity. IraD docks specifically to the N-terminal domain of RssB, IraP interacts primarily with the C-terminal domain, while IraM interacts with both domains. Despite these differences in binding, we propose that docking of each anti-adaptor induces a conformational change in RssB, which resembles the inactive dimer of RssB. This dimer-like state of RssB not only prevents substrate binding but also triggers substrate release from a pre-bound complex.

## Introduction

In their natural environment, bacteria are constantly exposed to changing and sometimes harsh environmental conditions. To survive these changes they have developed sophisticated stress response pathways to regulate the expression of specific genes that either restore cellular homeostasis or enable the bacteria to adapt to their new conditions. A key transcription factor or master regulator generally orchestrates these programmed changes. In *Escherichia coli* and related enteric bacteria, the cells response to a wide variety of different stress conditions (often referred to as the general stress response) is coordinated by a single transcription factor, σ^S^ (also known as σ^38^ or RpoS) (Hengge-Aronis, 2002). As such, the cellular levels of σ^S^ are highly regulated, not only at the transcriptional and translational levels, but also at the post-translational level through regulated proteolysis (Hengge, 2009; Battesti et al., 2011; Micevski and Dougan, 2013). In the absence of stress, σ^S^ levels remain low, largely as a result of its rapid turnover by the AAA+ (ATPases associated with a variety of cellular activities) protease ClpXP. Importantly, the turnover of σ^S^ is mediated by a specialized adaptor protein, RssB (also known as SprE) (Muffler et al., 1996; Pratt and Silhavy, 1996). RssB is a member of the response regulator (RR) family of proteins and like most RRs it is composed of two domains; a receiver domain and an effector (or output) domain (Galperin, 2010). However, in the case of RssB these domains have yet to be biochemically defined. As with most RRs the receiver domain of RssB is proposed to act as a phosphorylation-mediated switch, which regulates the activity of the effector domain (Gao and Stock, 2010). Consistent with this idea, RssB can be phosphorylated on a highly conserved Asp residue (Asp58). To date however, the physiological role of RssB-phosphorylation as a means to regulate σ^S^ levels remains controversial, as σ^S^ is only partially stabilized in cells carrying a non-phosphorylatable mutant of RssB (Peterson et al., 2004; Zhou and Gottesman, 2006). It has recently been proposed that cellular ATP levels directly control σ^S^ stability (Peterson et al., 2012). Independent of this control, both the recognition of σ^S^ (by RssB) and its degradation (by ClpXP) *in vitro* can be enhanced by phosphorylation of RssB. However, the mechanistic details by which RssB binds to σ^S^ and delivers it to ClpXP remains poorly understood (Hengge, 2009; Micevski and Dougan, 2013).

The stability of σ^S^ is also regulated by a group of unrelated proteins, which collectively have been termed anti-adaptors as they inhibit the activity of the adaptor protein RssB. Currently, three anti-adaptors have been identified in *E. coli*, all of which inhibit the turnover of σ^S^ in response to a specific stress (Bougdour et al., 2006, 2008). Related anti-adaptor proteins have also been identified in *Salmonella*, however despite their sequence similarity with *E. coli* anti-adaptors, these proteins are regulated by different stress conditions (Bougdour et al., 2008; Merrikh et al., 2009a,b). In *E. coli* the different anti-adaptors have been named, IraP (Inhibitor of RssB activity during phosphate starvation) which as the name suggests is specifically induced in response to phosphate starvation, IraD which is specifically induced in response to DNA damage and IraM which is specifically induced in response to magnesium starvation.

Although the transcriptional regulation of all three anti-adaptors has been extensively studied and is currently well understood (Bougdour et al., 2006, 2008; Bougdour and Gottesman, 2007; Merrikh et al., 2009a,b; Battesti et al., 2012), the mechanism of action of these proteins remains poorly defined. Here we show via a detailed biochemical analysis, that all three anti-adaptors use distinct modes of binding to inhibit RssB activity.

## Results

### RssB is composed of two domains

**Figure 1:**
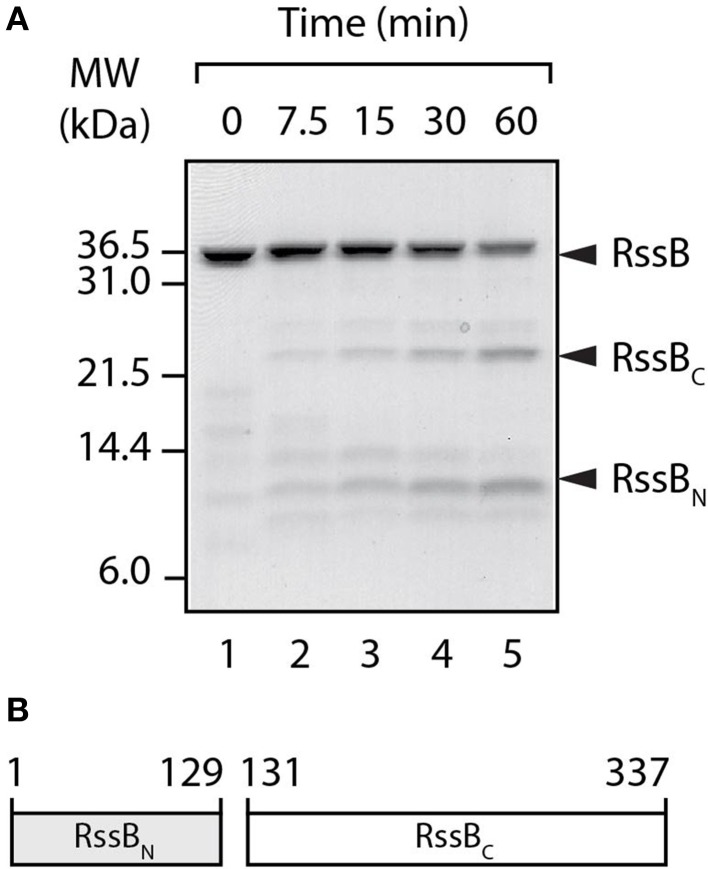
**RssB is composed of two domains. (A)** In the presence of thermolysin, RssB was digested into two stable fragments (RssB_N_ and RssB_C_). Proteins were separated by SDS-PAGE and stained with CBB. **(B)** Schematic diagram of recombinant RssB_N_ (residues 1–129) and RssB_C_, (residues 131–337) used in this study.

To dissect the mode of action of the three known anti-adaptor proteins, we first experimentally determined the domain boundaries of RssB. To do so, we performed limited proteolysis on untagged RssB (Figure 1). In the presence of thermolysin, untagged RssB (~36 kDa) was rapidly and specifically cleaved into two fragments (Figure 1A). N-terminal sequencing of the larger (~24 kDa) fragment (RVEEEE, corresponding to residues 131–136 of RssB) revealed this fragment to be the C-terminal effector domain. Therefore, in this study we defined the C-terminal domain of RssB (RssB_C_) as residues 131–337 and the N-terminal domain (RssB_N_) as residues 1–129 (Figure 1B). Next, untagged versions of each domain (RssB_N_ and RssB_C_) were purified (Figure S1A) using the ubiquitin (Ub) fusion system (Catanzariti et al., 2004). To assess the activity of these domains, a series of competition-based degradation experiments were performed, in which the RssB-mediated turnover of σ^S^ by ClpXP was monitored either in the absence or presence of RssB_N_ or RssB_C_ (Figure S1B). Importantly, neither RssB_N_ (Figure S1B, lower panel) nor RssB_C_ (Figure S1B, middle panel) altered the RssB-mediated turnover of σ^S^ (Figure S1B, upper panel). These findings validated the use of these domains to assess their ability to bind to and hence inhibit the activity of each anti-adaptor in competition assays.

### IraD binds to the N-terminal response regulator domain of RssB

**Figure 2:**
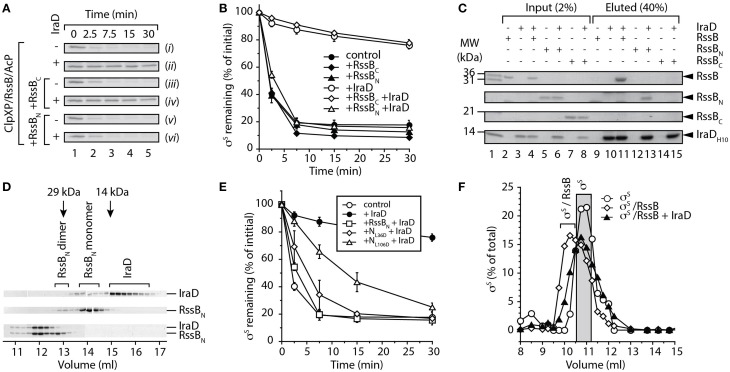
**IraD specifically interacts with the N-terminal domain of RssB. (A)** The RssB-mediated turnover of σ^S^ by ClpXP, was monitored in the absence of added domains (*i* and *ii*), or in the presence of either RssB_C_ (*iii* and *iv*) or RssB_N_ (*v* and *vi*), with (*ii*, *iv*, and *vi*) or without (*i*, *iii*, and *v*) IraD. Strips show σ^S^ following separation by SDS-PAGE and staining with CBB. **(B)** The RssB-mediated turnover of σ^S^ by ClpXP (control) was monitored either in the presence of RssB_C_ (diamonds) or RssB_N_ (triangles), with (open symbols) or without (filled symbols) IraD as shown in **(A)**. Quantification (using GelEval) of the relative amount of σ^S^ remaining, from three independent experiments (*n* = 3). Error bars represent the standard error of the mean (s.e.m.). **(C)** Ni-NTA agarose beads either lacking or containing immobilized IraD were incubated with either RssB, RssB_N_ or RssB_C_ (lanes 2–8) and following washing the bound proteins were eluted with imidazole (lanes 9–15). Proteins were separated by SDS-PAGE and visualized with CBB. **(D)** CBB-stained strips, from multiple SDS-PAGE gels showing fractions following separation of 5 nmol of either IraD (top panel), RssB_N_ (middle panel) or the IraD-RssB_N_ complex (lower panel), by SEC using Superdex 75. **(E)** The RssB-mediated turnover of σ^S^ by ClpXP (control) was monitored either in the absence (open circles) or presence (filled circles) of IraD. The IraD-inhibited turnover of σ^S^ was monitored in the presence of wild type (open squares), L36D (open diamonds) or L106D (open triangles) RssB_N_ (labeled RssB_N_, N_L36D_ and N_L106D_, respectively). Quantification (using GelEval) of the relative amount of σ^S^ remaining, from three independent experiments (*n* = 3). Error bars represent the s.e.m. **(F)** Following preincubation of σ^S^ with RssB (+AcP), IraD was added to the reaction prior to separation of the proteins using Superdex 200. Following separation of the proteins by SEC, the fractions were analyzed by SDS-PAGE and the amount of σ^S^ quantified using GelEval. The elution profile of σ^S^ (open circles) is shifted in the presence of RssB (open diamonds). The addition of IraD to a pre-formed RssB/σ^S^ complex prevents this shift of σ^S^ (filled triangles).

First we asked the question, does IraD interact with an individual domain of RssB? To address this question we monitored the ability of IraD to inhibit the RssB-mediated turnover of σ^S^, either in the absence of added domains [Figure 2A, (*ii*) and **B**, open circles] or in the presence of RssB_N_ [Figure 2A, (*vi*) and **B**, open triangles] or RssB_C_ [Figure 2A, (*iv*) and **B**, open diamonds]. As expected IraD was able to inhibit the RssB-mediated turnover of σ^S^, however in the presence of RssB_N_, the IraD-mediated inhibition of σ^S^ turnover was completely reversed. Importantly, this effect was specific for RssB_N_, as addition of RssB_C_ was unable to relieve IraD-mediated inhibition. These data indicate that only RssB_N_ can compete with full length RssB for binding to IraD and suggest that IraD binds specifically to the N-domain of RssB. To confirm if this relief of IraD inhibition on σ^S^ degradation was due to a specific interaction between IraD and the N-domain of RssB, a series of pull-down experiments were performed. In these experiments purified His_10_-tagged IraD was immobilized to Ni-NTA agarose beads and assessed for its ability to bind purified recombinant untagged RssB domains. As expected full-length RssB was specifically eluted from Ni-NTA beads containing immobilized IraD (Figure 2C, lane 11) and not from beads lacking immobilized protein (Figure 2C, lane 9). Importantly, only RssB_N_ was specifically eluted from beads containing immobilized IraD (Figure 2C, lane 13) confirming that the N-terminal domain of RssB is sufficient for interaction with IraD.

To validate this mode of binding and estimate the stoichiometry of the complex we performed size exclusion chromatography (SEC). In the absence of RssB_N_, IraD eluted in two peaks (Figure 2D, upper panel); the predominant peak (~15 ml) representing a monomer of IraD, and a minor peak (~14 ml) representing the IraD dimer. Similarly, in the absence of IraD, RssB_N_ also eluted in two peaks (Figure 2D, middle panel) a monomer at ~14 ml, and a minor dimeric peak (at ~13 ml). Importantly, when both proteins were incubated together and analyzed by SEC, both IraD and RssB_N_ (Figure 2D, lower panel) co-eluted, in a single peak at ~12 ml. Based on the molecular weight of this peak (estimated using protein standards) and the intensity of the two protein bands we propose that IraD and RssB_N_ form a heterodimer. Interestingly, a shift in the homodimer of RssB_N_ (albeit a minor component) was not observed, suggesting that this form of RssB_N_ is unable to interact with IraD (Figure 2D). To investigate a possible role of the RssB_N_ dimerization interface in IraD binding, we generated a mutant of RssB_N_ lacking the β5-α5 segment referred to here as RssB_N1−104_. Based on the structure of *Pseudomonas aeruginosa* RssB (PDB: 3EQ2; Levchenko et al., unpublished) this segment of RssB forms the dimerization interface. Analysis by SEC (data not shown) revealed that the elution profile of RssB_N1−104_ was not altered in the presence of IraD consistent with a loss of interaction. Collectively these data suggest that IraD binds to RssB through its dimerization interface. Importantly, the dimer of RssB is unable to recognize or deliver σ^S^ to ClpXP for degradation (Figure S2). Hence, we propose that IraD-binding, to the dimerization interface, triggers a switch in the conformation of RssB, which mimics the domain arrangement present in the dimeric form. We refer to this conformation as the “*off*” state.

To further validate this interaction site, we generated two single point mutations in RssB_N_, which based on its similarity to other RRs are located on opposite faces of the protein. In each case, a central hydrophobic residue (L36 within the N-terminal 1-2-2 interface or L106 within the C-terminal 4-5-5 interface) was replaced with aspartate (Figure S3). To monitor the effect of these single point mutations on the interaction with IraD we compared the ability of wild type and mutant N-domains (referred to here as N_L36D_ and N_L106D_, respectively) to relieve the IraD-mediated inhibition of σ^S^ degradation (Figure 2E). This analysis revealed that N_L106D_ exhibited a reduced ability to relieve the IraD-mediated inhibition of σ^S^ degradation in comparison to wild type RssB_N_ (Figure 2E, compare open triangles with open squares). In contrast, N_L36D_ retained the ability to inhibit IraD-activity (Figure 2E, open diamonds). Hence, these data confirm that IraD binds to N-terminal domain of RssB through the dimerization interface, which is directly linked to the C-terminal domain. Consequently, we propose that IraD-binding to the N-terminal domain may modulate communication to the C-terminal effector domain of RssB, where σ^S^ docking occurs (manuscript in preparation).

Next to determine if IraD was able to trigger release of σ^S^ from RssB we monitored the elution profile of the RssB/σ^S^ complex by SEC, before and after the addition of IraD (Figure 2F). In the absence of IraD, σ^S^ co-eluted with RssB in a single peak at ~10 ml (Figure 2F, open diamonds). However, following the addition of IraD to a preformed RssB/σ^S^ complex, the elution profile of σ^S^ (Figure 2F, filled triangles) resembled that of σ^S^ alone (Figure 2F, open circles). These data suggest that binding of IraD to the dimerization interface of RssB stabilizes the adaptor protein in a dimer-like conformation that triggers release of σ^S^.

### IraP binds to the C-terminal effector domain of RssB

**Figure 3:**
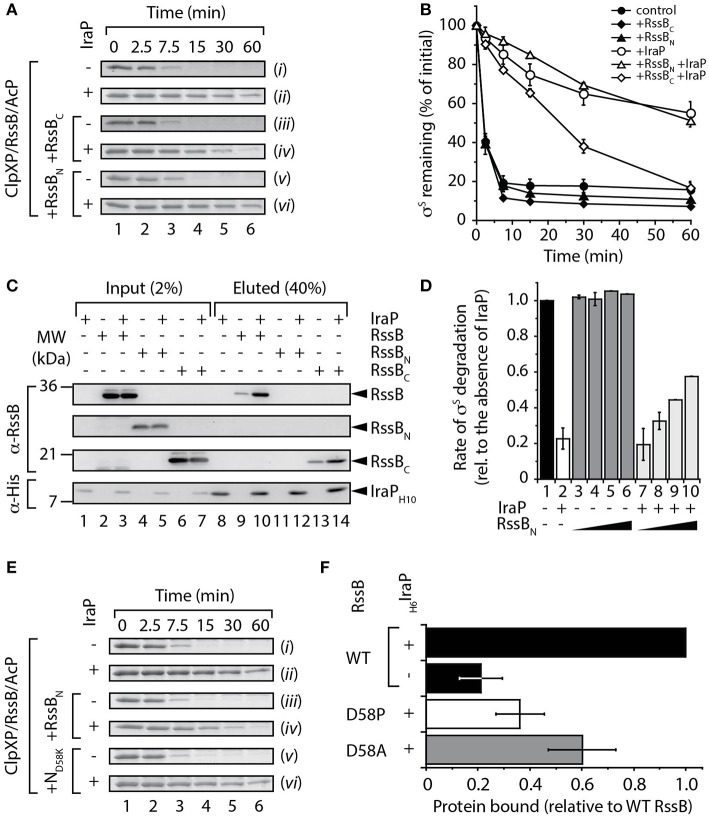
**Interaction of IraP with RssB. (A,B)** The RssB-mediated turnover of σ^S^ by ClpXP (control), was monitored in the absence of added domains (*i* and *ii*), or in the presence of either RssB_C_ (*iii* and *iv*) or RssB_N_ (*v* and *vi*), with (*ii*, *iv*, and *vi*) or without (*i*, *iii*, and *v*) IraP. Proteins were separated by SDS-PAGE and stained with CBB. Only the σ^S^-strip of the gel is shown. **(B)** The amount of σ^S^ remaining was quantified from three independent experiments. Error bars represent s.e.m. **(C)** Ni-NTA agarose beads either lacking or containing immobilized IraP were incubated with RssB, RssB_N_, or RssB_C_ (lanes 1–7) and following washing the bound proteins were eluted with imidazole (lanes 8–14). Proteins were detected by immunoblotting (as indicated) following separation by SDS-PAGE. **(D)** The initial rate of σ^S^ degradation in the absence of IraP (lane 1) was compared to the rate of degradation in the presence of IraP (lane 2) or with increasing concentrations (5, 10, 15, and 20 μM) of RssB_N_ either in the absence (lanes 3–6) or presence (lanes 7–10) of IraP. *n* = 3, and error bars represent the s.e.m. **(E)** The RssB-mediated turnover of σ^S^ by ClpXP, was monitored either in the absence of added domains (*i* and *ii*), or in the presence of wild type RssB_N_ (*iii* and *iv*) or N_D58K_ (*v* and *vi*), with (*ii*, *iv*, and *vi*) or without (*i*, *iii*, and *v*) IraP and separated by 15% SDS-PAGE. **(F)** The interaction of His_6_-IraP with wild type or mutant RssB was monitored by pull-down. Recovery of wild type RssB (black bar), D58P (white bar) or D58A (gray bar) was determined from the quantitation of three independent experiments. Error bars represent the s.e.m.

Next, we asked the question, does IraP use a similar or distinct mechanism to inhibit the RssB-mediated degradation of σ^S^? To address this question we monitored the ability of IraP to inhibit the RssB-mediated turnover of σ^S^, in the absence or presence of a 2.5-fold excess of either RssB_N_ or RssB_C_ (Figures 3A, B). Consistent with published data (Bougdour et al., 2006, 2008), recombinant IraP was able to inhibit the RssB-mediated degradation of σ^S^ by ClpXP [Figure 3A, compare (*ii*) with (*i*)], however in comparison to IraD, IraP was a less potent inhibitor of σ^S^ degradation (compare Figures 2B, 3B). Interestingly, in contrast to the analysis of IraD (Figure 2B, compare open diamonds with open circles) the addition of RssB_C_ was sufficient to partially inhibit IraP-activity as seen by the loss of inhibition of σ^S^ turnover (Figure 3B, compare open diamonds with open circles), while RssB_N_ had no effect on the turnover of σ^S^ (Figure 3B, compare open triangles with open circles). To examine more directly the mode of interaction between IraP and RssB, we monitored the binding of IraP with each RssB domain, using a series of pull-down experiments, in which purified recombinant IraP was immobilized to Ni-NTA agarose beads, via a C-terminal His_10_-tag (Figure 3C). Consistent with the competition degradation assays (Figures 3A, B), an interaction between RssB_C_ and immobilized IraP was observed (Figure 3C, lane 14). Although a specific interaction between RssB_N_ and IraP was not detected in most pull-down experiments as shown in the representative example (Figure 3C, lane 12) “weak” binding was observed in a single experiment. Collectively, these data clearly indicate that IraP docks onto the C-terminal effector domain of RssB however a potential role, for the N-terminal domain could not be completely excluded. To further examine any possible contribution of the N-terminal domain of RssB in binding to IraP, we again employed the competition-based degradation assay to monitor σ^S^ turnover. In this case, the turnover of σ^S^ was monitored in the presence of increasing concentrations of RssB_N_ (up to a 20-fold excess), either in the absence or presence of IraP (Figure 3D). Consistent with a weak interaction between IraP and RssB_N_ the rate of σ^S^ turnover (in the presence of IraP) increased in the presence of higher concentrations of RssB_N_ (Figure 3D, columns 7–10), while the same concentrations of RssB_N_ did not affect the rate of σ^S^ turnover in the absence of IraP (Figure 3D, columns 3–6). Collectively these data suggest that in contrast to IraD, IraP interacts with both domains of RssB. However, based on the relative ability of each domain to inhibit IraP activity, IraP appears to bind with higher affinity to the C-terminal domain of RssB.

Given the apparently weak interaction of IraP with the N-terminal domain, we chose to further clarify this interaction by examining the role of phosphorylation (of Asp58) on IraP-binding. In this case, we initially generated a non-phosphorylatable mutant of RssB_N_ in which Asp58 was replaced with Lys (here referred to as N_D58K_) and compared the ability of this mutant (relative to wild type RssB_N_) to inhibit IraP binding to full-length RssB in the presence of the phospho-donor, AcP (Figure 3E, S4). Importantly, given that this mutant cannot be phosphorylated, this permitted a direct comparison of RssB_N_ phosphorylation and IraP binding in the presence of AcP. Consistent with our previous findings (Figure 3D), a 5-fold excess of RssB_N_ resulted in a ~2-fold increase in the rate of σ^S^ turnover in the presence of IraP (Figure S4). In contrast, a 5-fold excess of N_D58K_ did not alter the IraP-mediated inhibition (Figure 3E, S4). Next we performed a series of pull-down experiments to directly monitor the interaction between IraP and RssB (Figure 3F). In this case, we replaced Asp58 with either Pro or Ala to generate two well-characterized non-phosphorylatable mutants of RssB, RssB(D58P) or RssB(D58A) (Bouche et al., 1998; Peterson et al., 2004), here referred to as D58P and D58A, respectively. Specifically, wild type or mutant RssB was expressed in presence or absence of His_6_-tagged IraP, the cell lysate was then applied to Ni-NTA beads and after extensive washing to remove non-specifically bound proteins, IraP was eluted with imidazole and the relative amount of RssB (wild type or mutant) specifically co-eluting with IraP determined. Consistent with the *in vitro* pull-down (shown in Figure 3C) a small amount of RssB was found to interact non-specifically with the beads however the levels of RssB recovered were significantly increased in the presence of IraP (Figure 3F, compare black bars). Importantly, mutation of Asp58 (to either Pro or Ala) resulted in a dramatic loss in the amount of RssB bound to IraP (Figure 3F, white or gray bars, respectively). Collectively, these data suggest that IraP docks to the C-terminal domain of RssB, however its binding appears be stabilized by the phosphorylation state of the N-terminal domain. Next, to confirm if the stabilized binding of IraP to RssB_C_ was driven by a conformational change in the C-terminal domain of RssB induced by phosphorylation of the N-domain we examined if binding of RssB_C_ (or indeed RssB_N_) could be enhanced *in trans* (Figure S5). To do so, we monitored the binding of IraP to each RssB domain, either alone or together (Figure S5). Consistent with the idea that phosphorylation of the receiver domain drives a conformational change in the effector domain, which triggers improved interaction with IraP, the addition of RssB_N_ *in trans* did not enhance the recovery of RssB_C_. In contrast, the recovery of RssB_N_ was reduced in the presence of RssB_C_, confirming that the N-domain alone interacts only weakly with IraP (Figure S5, lane 5).

### IraM binds to both domains of RssB

**Figure 4:**
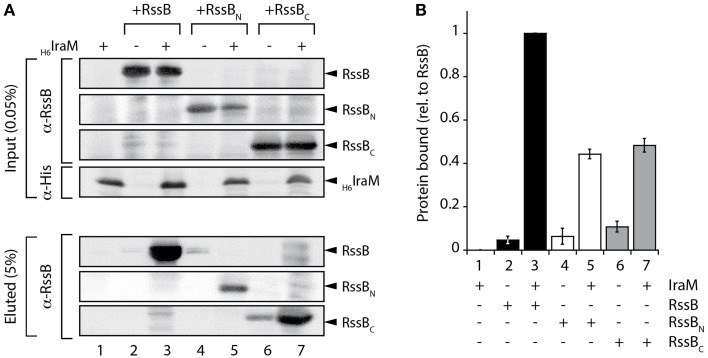
**Interaction of IraM and RssB. (A)** Following incubation of Ni-NTA agarose beads with lysates either lacking (lanes 2, 4, and 6) or containing (lanes 1, 3, 5, and 7) overexpressed His_6_-IraM, in the presence of recombinant RssB (lanes 2 and 3), RssB_N_ (lanes 4 and 5) or RssB_C_ (lanes 6 and 7) the beads were washed extensively then IraM, together with interacting proteins were eluted with imidazole. Proteins were detected by immunoblotting (as indicated) following separation by 16.5% Tris-Tricine SDS-PAGE. **(B)** The recovery of RssB_N_ (white bar) or RssB_C_ (gray bar) relative to RssB (black bar) was determined from the quantitation of three independent experiments. Error bars represent the s.e.m.

To determine if the third anti-adaptor, IraM, mimicked the action of IraD or IraP, its ability to bind to the domains of RssB was examined. In this case, due to poor recovery of recombinant IraM, we chose to monitor the binding of IraM to the different domains of RssB via pull-down experiments using cell lysates in which the proteins of interest were co-expressed. Specifically, we generated a series of clones, which enabled the overexpression of untagged RssB (full-length or individual domains) in the absence or presence of overexpressed His_6_-IraM. Following preliminary evaluation of the levels of soluble IraM and RssB (within the cell lysate) via SDS-PAGE (Figure S6) the appropriate amount of each lysate was applied to Ni-NTA agarose beads and the pull-down performed. The eluted proteins were evaluated by immunoblotting (Figure 4A) using the appropriate antibody. As expected, full-length RssB was specifically recovered, from the column containing immobilized His_6_-IraM (Figure 4A, lane 3). Interestingly, in contrast to both IraD and IraP, IraM appeared to form a stable interaction with both domains of RssB (Figure 4A). Indeed, based on the recovery of each domain in comparison to the input, both domains appear to contribute equally to IraM binding (Figure 4B). These data indicate that IraM imposes its inhibitory effect on RssB activity through docking to both domains of RssB. Although this mode of binding is similar to that of IraP, the relative contribution of each domain appears to be quite different. Next, we examined if both domains can function *in trans*. To do so we compared the binding of each domain to IraM, either alone or in the presence of the other domain (Figure S7). Unexpectedly, and in contrast to IraP (Figure S5), we observed an improved recovery of RssB_N_ (when incubated in the presence of RssB_C_). These data suggest that the C-terminal domain of RssB can promote binding of RssB_N_ to IraM, by stabilizing RssB_N_ (or IraM) in a conformation that is permissive for interaction with the other component. Collectively, these data suggest that all three anti-adaptors use distinct modes of binding to inhibit RssB-activity.

### Anti-adaptor binding triggers a conformational change in RssB

**Figure 5:**
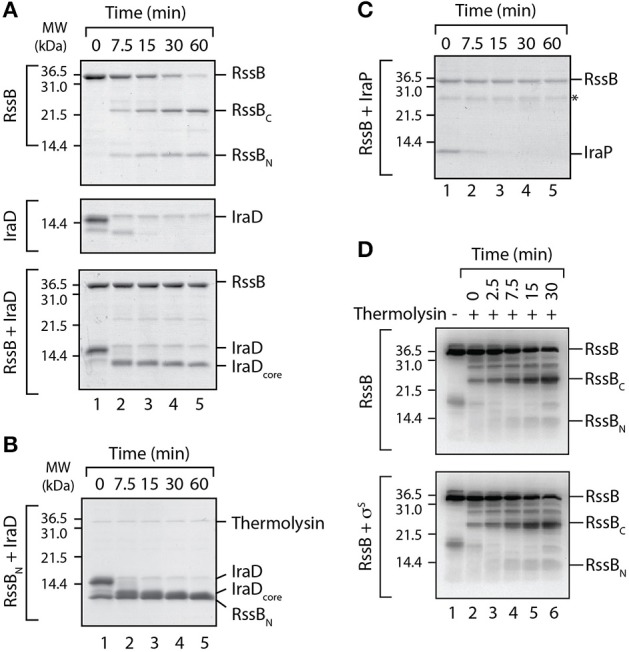
**IraD and IraP trigger a conformational change in RssB. (A)** The domain structure of RssB was analyzed by partial proteolysis using thermolysin in the absence (top panel) or presence (lower panel) of IraD. As a control IraD (alone) was also analyzed in the presence of thermolysin (middle panel). **(B)** The core domain of IraD (IraD_core_) is stabilized by RssB_N_ (from partial proteolysis with thermolysin). **(C)** The domain structure of RssB was monitored in the presence of IraP, using thermolysin. **(A–C)** Proteins were separated by SDS-PAGE and stained with CBB. **(D)** SigmaS (σ^S^) is unable to protect the RssB linker region from cleavage by thermolysin. The domain structure of RssB was analyzed by partial proteolysis with thermolysin in the absence (top panel) or presence (bottom panel) of σ^S^. Following partial proteolysis in the presence of thermolysin (lanes 2–6), the protein fragments were separated by SDS-PAGE, transferred to PVDF membrane and immunodecorated with α-RssB antisera, to specifically detect the RssB fragments.

While each anti-adaptor exhibited a different mode of binding to RssB it still remained to be determined how they inhibit the RssB-mediated recognition of σ^S^. One possibility is that each anti-adaptor simply inhibits substrate engagement by RssB via steric hindrance due to the presence of the bound anti-adaptor. This however, at least in the case of IraD, appears unlikely as the anti-adaptor binds specifically to RssB_N_ and this binding is sufficient to trigger release of σ^S^ from the C-domain of RssB, in a preformed RssB/σ^S^ complex. An alternative possibility is that anti-adaptor binding triggers a conformational change in RssB that promotes release of σ^S^ from a second site. To gain further insight into the mode of action of the RssB anti-adaptors we repeated the partial proteolysis experiments, in the absence or presence of either IraD (Figure 5A) or IraP (Figure 5C). Consistent with our previous data (Figure 1A), in the absence of anti-adaptor, RssB was rapidly cleaved into two stable domains upon addition of thermolysin (Figure 5A, top panel). In contrast to RssB, IraD was rapidly and completely degraded following the addition of thermolysin (Figure 5A, middle panel). However, when IraD was incubated with RssB, both proteins were clearly protected from cleavage by thermolysin (Figure 5A, lower panel). In the case of RssB, the entire protein was completely stable while in the case of IraD only a fragment of the protein (here termed IraD_core_) remained stable throughout the experiment. To determine if stabilization of the IraD_core_ fragment was solely due to its interaction with the N-terminal domain of RssB, we repeated the IraD partial proteolysis experiment in the presence of RssB_N_ (Figure 5B). These data clearly show, that RssB_N_ is not only sufficient to stabilize the core fragment of IraD, but also verifies that IraD_core_ migrates with a different mobility to RssB_N_. Collectively these data suggest that binding of IraD to the N-terminal domain of RssB is sufficient to trigger a conformational change in RssB, which protects it from cleavage by thermolysin. Next we examined whether IraP also protected RssB in the limited proteolysis assay, in the same manner as IraD. Consistent with the effect of IraD, IraP also stabilized full-length RssB, however in contrast to IraD, a concomitant stabilization of the anti-adaptor was not observed (Figure 5C). These data suggest that only a short fragment of IraP is required for binding to RssB, and that this fragment is sufficient to stabilize the protease-protected or inactive conformation of RssB. Importantly, in contrast to both IraD and IraP, σ^S^ did not protect RssB from cleavage by thermolysin (Figure 5D). These data suggest that in comparison to anti-adaptor docking, substrate binding to RssB either occurs through a different site on RssB or alternatively does not trigger the same conformational change.

## Discussion

RpoS is the central regulator of the general stress response in *E. coli*. In the absence of stress, the cellular levels of σ^S^ are undetectable (< 1 molecule/cell), however in the presence of stress the level of σ^S^ increases rapidly, up to ~ 230 molecules/cell during stationary phase (Jishage and Ishihama, 1995; Maeda et al., 2000). This rapid change in cellular concentration of σ^S^ is largely achieved by regulating its turnover. Under normal conditions σ^S^ is rapidly degraded by the AAA+ protease ClpXP, which is mediated by a specialized adaptor protein RssB. However, upon exposure to stress the RssB-mediated turnover of σ^S^ is inhibited by one of three stress-specific anti-adaptor proteins IraD, IraP or IraM. Although the molecular components of this regulated protein degradation pathway have been reconstituted *in vitro*, currently little progress has been made toward determining the molecular basis of inhibition by these anti-adaptors.

**Figure 6:**
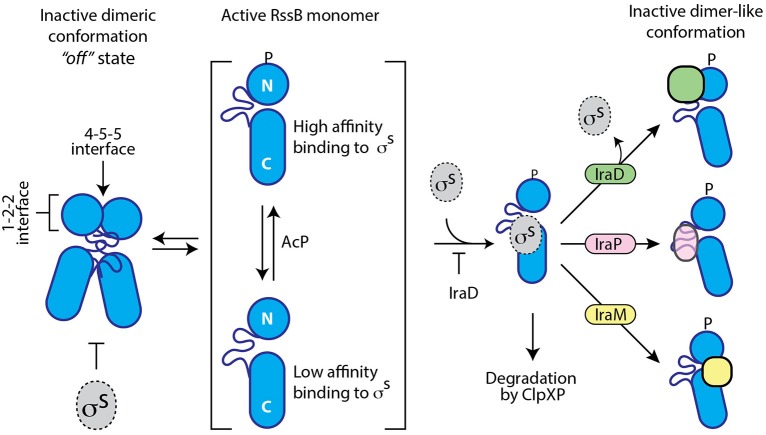
**Cartoon illustrating a model of the different modes of action of the three anti-adaptors, IraD (green), IraP (pink), and IraM (yellow)**. In the absence of anti-adaptors, RssB (blue) is able to recognize the transcription factor σ^S^ (gray). This recognition is inhibited by dimerization of RssB and enhanced by phosphorylation of the N-terminal domain of RssB. Anti-adaptor binding not only inhibits the recognition of σ^S^, but in the case of IraD, also triggers the release of σ^S^. Binding of IraD (to RssB_N_) and IraP (to RssB_C_) triggers a conformational change in RssB, which we propose resembles the dimer-like state or RssB. Binding of IraM to both domains inhibits σ^S^ interaction via an unknown mechanism.

Here, we have experimentally determined the domain structure of RssB (Figure 1) and defined the first mechanistic details of each anti-adaptor. Interestingly, each anti-adaptor interacts with RssB in a specific manner. Consistent with recent data from Gottesman and colleagues (Battesti et al., 2013) we found that IraD docks exclusively to the N-terminal domain (Figure 2) and IraM interacts with both domains (Figure 4). However, in contrast to Gottesman and colleagues (Battesti et al., 2013), we find that IraP interacts primarily with the C-terminal domain (Figure 3) although the interaction appears to be modulated by phosphorylation of the N-domain of RssB. Importantly, our data suggests that anti-adaptor docking, to either domain, triggers a conformation change in RssB (Figure 5), which results in substrate release. Specifically, we propose that IraD binding, to the dimerization interface of RssB, stabilizes an inactive conformation of RssB, which triggers release of σ^S^ from the C-terminal effector domain. Consistent with this model, binding of IraP to the C-terminal domain of RssB appears to induce a similar conformational change and hence we speculate that all three anti-adaptors, independent of their mode of docking to RssB, induce a conformational change in RssB, that mimics the inactive dimer-like state of RssB, which is unable to bind (or deliver) σ^S^ (this model is summarized in Figure 6).

In the case of IraP, a short region of this anti-adaptor appears to be sufficient to stabilize the inactive conformation of RssB. Consistent with this idea, a short region (hexapeptide) of the *Bacillus subtilis* anti-adaptor–ComS, is sufficient to displace the substrate ComK from the adaptor protein MecA, and hence inhibit its degradation by the ClpCP protease (Turgay et al., 1998; Prepiak and Dubnau, 2007). In this case however, the anti-adaptor appears to compete directly with the substrate for binding to the same site on MecA and as a result ComS is also degraded by the ClpCP/MecA protease. In contrast to MecA, we propose that RssB interacts with the anti-adaptors (IraD and IraP) and the substrate (σ^S^) through distinct binding sites, as degradation of the RssB-anti-adaptors by their cognate protease has not been observed.

Based on bioinformatic analysis of the IraP amino acid sequence, the short region of IraP responsible for interaction with RssB could be located within the N-terminus of the anti-adaptor, as this region is predicted to form a coiled-coil (residues 1–35). Similarly a short segment (residues 127–155) in the C-terminal domain of RssB is also predicted to form a coiled-coil. Given that coiled-coils often play important roles in biological interactions, one possibility is that both coiled-coils drive the formation of the heterodimeric complex. This interaction could result in a conformational change to RssB, or alternatively it could stabilize a conformation of RssB, which prevents substrate binding. Consistent with this idea, a single point mutant (L9S) within the coiled-coil region of IraP reduces its inhibitory activity (Bougdour et al., 2006). Interestingly, the structure of full-length RssB from *P. aeruginosa* (3EQ2) also contains a coiled-coil region, located between the N- and C-terminal domains, which appears to mediate homodimer formation. Based on our biochemical data, the dimeric conformation of *E. coli* RssB is unable to deliver σ^S^ to ClpXP for degradation. Therefore, we propose that IraP, similar to IraD, stabilizes RssB in a dimer-like conformation, which renders RssB unable to recognize or deliver σ^S^ to ClpXP for degradation. This mode of inhibition shares some striking similarities to the regulation of a quorum sensing transcriptional activator (TraR) in bacteria such as *Agrobacterium tumefaciens*, which is controlled through its interaction with a quorum sensing anti-activator, known as TraM. Similar to IraP, TraM also contains a coiled-coil region, which is involved not only in homodimer formation (Chen et al., 2004), but also in binding to TraR resulting in the formation of the TraR-TraM anti-activation complex (Chen et al., 2007). In this case, TraM binding to one site in TraR, has been shown to cause a conformational change in TraR that prevents substrate binding at a second site.

Collectively, our data shows that each anti-adaptor forms a stable complex with RssB, albeit through a unique mode of binding. Atomic resolution structures of each anti-adaptor, both alone and in complex with the adaptor protein RssB, are eagerly awaited to better understand the nature of these interactions.

## Experimental procedures

### Protein purification and size exclusion chromatography

His_6_-tagged ClpX and ClpP were overexpressed in *E. coli* and purified as described previously (Dougan et al., 2003). His_10_-tagged IraP and IraD were overexpressed in *E. coli* and purified essentially as described by Bougdour et al. (2008). Untagged σ^S^, RssB, RssB_C_, and RssB_N_ (wild type and specific point mutants) were generated using the Ub-fusion system (Catanzariti et al., 2004) and purified essentially as described previously (Ninnis et al., 2009), using a combination of IMAC and preparative grade SEC to separate monomeric and dimeric RssB. To examine the different protein complexes, analytical SEC was performed. All columns were pre-equilibrated in chilled GF buffer (20 mM Tris-HCl pH 7.5, 10 mM MgCl_2_, 0.1 mM EDTA, 1 mM DTT, 140 mM NaCl, 5% (v/v) glycerol, 0.005% (v/v) Triton X-100).

### *In vitro* degradation assays

The *in vitro* σ^S^ degradation assays were performed essentially as described (Zhou et al., 2001) with minor modifications. All reactions were performed in degradation buffer (20 mM Tris-HCl pH 7.5, 140 mM NaCl, 10 mM MgCl_2_, 0.1 mM EDTA, 5% (v/v) glycerol, 0.005% (v/v) Triton X-100, 1 mM DTT) and contained 1 μM σ^S^. Unless otherwise stated reactions containing σ^S^ were pre-incubated at 30°C, with 1 μM ClpX, 1 μM ClpP and 0.08 μM monomeric RssB and 20 mM of the phospho donor, acetyl phosphate (AcP) (Bouche et al., 1998). Where indicated IraD (1 μM), IraP (1 μM), RssB_N_ (2.5–20 μM), and RssB_C_ (2.5 μM) were included. All reactions were initiated with the addition of 2 mM ATP and samples collected at the specified time-points. Samples were separated using SDS-PAGE and visualized using Coomassie Brilliant Blue (CBB) staining.

### Limited proteolysis

Limited proteolysis experiments were performed using thermolysin as described previously (Lowth et al., 2012). Following a short pre-incubation (2 min at 25°C) in the absence of the protease, the substrate (i.e., RssB with or without anti-adaptor) was incubated with the protease. Reactions were stopped with the addition of PMSF (6 mM) and 1 × SDS-PAGE sample buffer. Proteins were separated by SDS-PAGE and visualized by staining with CBB.

### *In vitro* “pull-down” experiments

To examine the interaction of RssB (either full-length protein or individual domains) with purified IraD or IraP, *in vitro* “pull-down” experiments were performed as previously described (Geissler et al., 2002). To examine the interaction of IraM or IraP with RssB (full-length protein (wild type or mutant) and individual domains), “pull-down” experiments were performed using soluble protein extracts isolated from BL21(DE3) codon+ cells in which untagged RssB (full-length (wild type or mutant) or individual domains) was overexpressed in the absence or presence of either His_6_-IraM or His_6_-IraP. Following overexpression of the proteins, a series of soluble lysates were prepared in extraction buffer (20 mM Tris-HCl pH 7.9, 500 mM NaCl, 10% (v/v) glycerol, 10 mM imidazole, 1% (v/v) Triton X-100). Based on the amount of IraM present within each soluble lysate, between 40 and 160 mg of total protein was applied to 100 μl of pre-equilibrated Ni-NTA agarose beads. To monitor the *in trans* binding of each RssB domain to the immobilized anti-adaptor proteins (IraP or IraM) the appropriate lysate was supplemented with 250 μg of RssB_N_. Soluble protein lysates (in the presence or absence of additional RssB_N_) and Ni-NTA agarose beads were then incubated for 30 min at 4°C. The beads were then transferred to individual MoBiTec columns. Further washes and recovery of bound proteins was performed as described (Geissler et al., 2002).

### Conflict of interest statement

The authors declare that the research was conducted in the absence of any commercial or financial relationships that could be construed as a potential conflict of interest.
