# Supplementary material for: Anti-adaptors use distinct modes of binding to inhibit the RssB-dependent turnover of RpoS (σS) by ClpXP
Source: Front Mol Biosci. 2015 Apr 23;2:15. doi: 10.3389/fmolb.2015.00015 (PMC4428439; doi:10.3389/fmolb.2015.00015)
Supplement: Supplementary file 1 [file Presentation1.PDF]

Supplementary Experimental procedures

Cloning

Genes of interest were amplified from *E. coli* genomic DNA by polymerase chain reaction (PCR), using specifically designed synthetic oligonucleotide primers each containing an appropriate restriction endonuclease site (see Table S1) and cloned into a suitable *E. coli* expression vector. For specific details regarding the construction of each plasmid refer to Table S2. To generate single point mutations in specific genes, site directed mutagenesis was performed using a pair of partially complimentary primers that annealed to the target DNA (see Table S1) as described (Zheng *et al.*, 2004). All positive clones were screened by digestion with the appropriate restriction enzymes and verified by nucleotide sequencing.

N-terminal sequencing (Edman degradation)

Protein fragments were separated by SDS-PAGE, transferred to a PVDF membrane in CAPS buffer (10 mM 3-[cyclohexylamino]-1 propane sulfonic acid (CAPS) pH 11, 10% (v/v) methanol), then stained using CBB. Following staining, the appropriate band (~30 pmol) was excised from the membrane and 5 cycles of Edman degradation performed by the Australian Proteome Analysis Facility (APAF), using an Applied Biosystems 494 Procise Sequencing system.

Antibodies (and detection)

Polyclonal antibodies against *E. coli* RssB were raised in rabbit using purified recombinant thioredoxin-tagged full length RssB as the antigen. Following western transfer using a semi-dry system, the protein of interest (RssB, $RssB_N$ and $RssB_C$) was immunodecorated with either α-RssB antisera (1:1000 – 1:10000) or α-His antibodies (1:2000) followed by a horseradish peroxidase-coupled secondary antibody (either goat-α-rabbit or goat-α-mouse, respectively) and detected using enhanced chemiluminescence reagents (GE Healthcare). Imaging was performed

using ChemiGenius2 Bio-Imaging System (SynGene) and the digital images captured using GeneSnap software (SynGene).

References

Zheng L, Baumann U, & Reymond JL (2004) An efficient one-step site-directed and site-saturation mutagenesis protocol. *Nucleic Acids Res* **32**: e115

Supplementary figure legends

Fig. S1. RssB is composed of two domains. (*A*) CBB stained 15% SDS-PAGE showing purity of recombinant $RssB_N$ (lane 1) and $RssB_C$ (lane 2), which migrate with the same mobility as the fragments of full length RssB, generated by partial proteolysis using thermolysin (lanes 4 – 9). M12 protein standards (lane 3). (*B*) The RssB-mediated turnover of σ^S by ClpXP, was monitored in the absence of added domains (upper panel) or in the presence of either $RssB_C$ (middle panel) or $RssB_N$ (lower panel). Strips show σ^S following separation by SDS-PAGE and staining with CBB.

Fig. S2. Dimeric RssB exhibits a greatly reduced ability to interact with and deliver σ^S to ClpXP for degradation. Following purification of "*momomeric*" and "*dimeric*" RssB by SEC, the respective proteins were immediately added to a σ^S degradation assay in the presence (lanes 1 – 5) or absence (lanes 6 - 10) of AcP. Samples were collected at the indicated time points and separated by 15% SDS-PAGE, before staining of proteins with CBB, strips show the stained band corresponding to σ^S.

Fig. S3. Structure of *E. coli* RssB N-terminal domain (PDB: 3EOD) visualized using UCSF Chimera. Ribbon representation of RssB N-terminal domain (grey) showing the distal interface (left panel) including residue L36 (highlighted in blue) and the dimerization interface (right panel) including residue L106 (highlighted in blue).

Fig. S4. The initial rate of σ^S degradation (from Fig. 3E), in the absence of IraP (columns 1, 3 and 5) was compared to the rate of degradation in the presence of IraP (columns 2, 4 and 6), either without (columns 1 and 2) or with (columns 3 and 4) $RssB_N$ or with N_{D58K} (columns 5 and 6).

Fig. S5. Representative pull-down of $RssB_C$ in the presence or absence of $RssB_N$

Following incubation of Ni-NTA agarose beads with lysates either lacking (lanes 3) or containing (lanes 1, 2, 4 and 5) overexpressed His_6-IraP, in the presence of overexpressed $RssB_C$ (lanes 3 - 5) with the addition of 250 μg of $RssB_N$ (lanes 2 and 5), the beads were washed extensively, then IraP together with interacting proteins was eluted with imidazole. RssB domains were detected by immunoblotting following separation by 16.5% Tris-Tricine SDS-PAGE. * represents a non-specific immunoreactive band.

Fig. S6. Relative levels of soluble H_6-IraM and RssB expression. The expression levels of H_6-IraM (and full-length RssB and domains) were analyzed by CBB staining of proteins separated by 15% SDS-PAGE, before (- IPTG) and after (+ IPTG) induction of H_6-IraM (lanes 2 and 3), RssB (lanes 4 and 5), H_6-IraM in the presence of RssB (lanes 6 and 7), $RssB_N$ (lanes 8 and 9), H_6-IraM in the presence of $RssB_N$ (lanes 10 and 11), $RssB_C$ (lanes 12 and 13) and H_6-IraM in the presence of $RssB_C$ (lanes 14 and 15). M12 protein standards (lane 1).

Fig. S7. Interaction of IraM and RssB.

A. Following incubation of Ni-NTA agarose beads with lysates either lacking (lanes 1 and 3) or containing (lanes 2, 4 and 5) overexpressed His_6-IraM, in the presence of either overexpressed $RssB_N$ (lanes 1 and 2) or $RssB_C$ (lanes 3 – 5) with the addition of 250 µg of $RssB_N$ (lane 5), the beads were washed extensively, then IraM together with interacting proteins were eluted with imidazole. RssB domains were detected by immunoblotting following separation by 16.5% Tris-Tricine SDS-PAGE.

B. The recovery of $RssB_N$ (grey bars) or $RssB_C$ (white bars) was determined relative to the control (i.e. lane 2 for $RssB_N$ and lane 5 for $RssB_C$) from the quantitation of three independent experiments. Error bars represent the s.e.m.

Table S1 Oligonucleotide primers used in this study

Primer	*Oligonucleotide sequence (5'-> 3')*	*Gene*	*Features*
5Nde_iraD	CGATGACATATGATGCGACAATCAC TTCAGGC	*iraD*	*Nde* I *restriction site for cloning into pET10C*
3iraD_not	CATGACTGCGGCCGCGCTGACATTC TCCAGCGTCGC	*iraD*	*Not* I *restriction site for cloning into pET10C*
5iraP_10C	GACTCTCATATGAAAAATCTCATTG CTGAG	*iraP*	*Nde* I *restriction site for cloning into pET10C*
3iraP_10C	TATGATGCGGCCGCCTGACGAGGAT GCTTC	*iraP*	*Not* I *restriction site for cloning into pET10C*
bam_iraP	CTGATCGGATCCGAAAAATCTCATT GCTGAGTTG	*iraP*	*Bam* HI *restriction site for cloning into pETduet-1*
iraP_not	TATGATGCGGCCGCTTACTGACGAG GATGCTTC	*iraP*	*Not* I *restriction site for cloning into pETduet-1*
5bam_iraM	GACTCTGGATCCAAAGTGGATAGTA ATTGACAC	*iraM*	*Bam* HI *restriction site for cloning into pETduet-1*
3iraM_hind	ATAGCGAAGCTTACTTGAGCCCATA TGGGC	*iraM*	*Hind* III *restriction site for cloning into pETduet-1*
5Ub-sigS	GACTCTCCGCGGTGGAAGTCAGAAT ACGCTGAAAGTTC	*rpoS*	*Sac* II *restriction site for cloning into pHUE*
3sigS_hind	TGGTCGAAGCTTACTCGCGGAACAG CGC	*rpoS*	*Hind* III *restriction site for cloning into pHUE*
RssB_bam	GACTCTGGATCCATGACGCAGCCAT TGGTCGG	*rssB*	*Bam* HI *restriction site for cloning into pHUE*
RssB_hind	TATGATAAGCTTCATTCTGCAGACA ACATCAAG	*rssB*	*Hind* III *restriction site for cloning into pHUE*
RssB_N1	CCCAGCATGTAAGGATCCCGCGTTG AGGAAGAGGAAAGG	*rssB*, $rssB_C$	*Introduce Bam* HI *restriction site to create* $RssB_N$ *in pHUE, pET32 or pETduet-1 and* $RssB_C$ *in pHUE*
RssB_N2	CCTCAACGCGGGATCCTTACATGCT GGGATAGAGACAGG	*rssB*, $rssB_C$	*Introduce Bam* HI *restriction site to create* $RssB_N$ *in pHUE, pET32 or pETduet-1 and* $RssB_C$ *in pHUE*
RssB_shN1	CGTTGAAGATTAATTGCTGAAACCA GTTAAAG	$rssB_N$	*Introduce stop codon, to generate* $rssB_{N1\text{-}104}$ *in pHUE*
RssB_shN2	GTTTCAGCAATTAATCTTCAACGCC CAGACG	$rssB_N$	*Introduce stop codon, to generate* $rssB_{N1\text{-}104}$ *in pHUE*
Nde_rssB	TATCGCCATATGACGCAGCCATTGG TCGGAAAAC	*rssB*	*Introduce Nde* I *restriction site to create RssB in pETduet-1*
Nde_rssBc	TATCGCCATATGTTTAATTCGCGCG TTGAGG	$rssB_C$	*Introduce Nde* I *restriction site to create* $RssB_C$ *in pETduet-1*
Nco_rssBc	TAGATCCCATGGTGTTTAATTCGCG CGTTG	$rssB_C$	*Introduce Nco* I *restriction site to create* $RssB_C$ *in pET32*
rssB_stop_xho	CATCAGCTCGAGTCATTCTGCAGAC AACATC	$rssB_C$	*Introduce Xho* I *restriction site to create* $RssB_C$ *in pET32 or pETduet-1*
D58A_1	ATCGCGATGCCACGAATGAACGGGC TTAAAC	*rssB*	*Introduce D58A mutation, introduces Pvu* I *restriction site*
D58A_2	CGCACATATCATCAGGTCTGGAGTG AAACC	*rssB*	*Introduce D58A mutation, introduces Pvu* I *restriction site*
D58K_1	GATGATATGTAAAATCGCGATGCCA CGAATG	*rssB*	*Introduce D58K mutation, removes Eco* RV *restriction site*
D58K_2	CATCGCGATTTTACATATCATCAGG TCTGGAG	*rssB*	*Introduce D58K mutation, removes Eco* RV *restriction site*

D58P_1	ATGCCgCGgATGAACGGGCTTAAACTGCTGG	*rssB*	*Introduce D58P mutation, introduces Sac* II *restriction site*
D58P_2	CGCGATcggACATATCATCAGGTCTGGAGTG	*rssB*	*Introduce D58P mutation, introduces Sac* II *restriction site*
L36D_1	GACAACGGTATGCGCGACAGATGGGGTGGATGC	*rssB*	*Introduce L36D mutation*
L36D_2	CCCATCTGTCGCGCATACCGTTGTCGCTCCCAATG	*rssB*	*Introduce L36D mutation*
L106D_1	GAAGATGTTGACCTGAAACCAGTTAAAGATCTG	*rssB*	*Introduce L106D mutation*
L106D_2	CTGGTTTCAGGTCAACATCTTCAACGCCCAGACG	*rssB*	*Introduce L106D mutation*

[1] restriction sites for cloning and/or screening are underlined

Table S2 Plasmids used in this study

**Name**	**Description**		**Source**
	gene	plasmid	
pDT2102	*rssB*	pHUE	Amplified *rssB* using RssB_bam and RssB_hind, digested with *Bam* HI and *Hind* III and cloned into pHUE
pDT2134	*rssB*$_N$	pHUE	Quick change mutagenesis using pDT2102 and primers RssB_N1 and RssB_N2
pDT2149	*rssB*$_C$	pHUE	Quick change mutagenesis using pDT2102 and primers RssB_N1 and RssB_N2, digestion with *Bam* HI to remove the fragment coding for *rssB*$_N$ followed by ligation of digested plasmid
pDT2369	*rssB*$_{N1-104}$	pHUE	Quick change mutagenesis using pDT2134 and primers RssB_shN1 and RssB_shN2.
pDT2467	*iraM*	pETduet-1	Amplified *iraM* using 5bam_iraM and 3iraM_hind, digested with *Bam* HI and *Hind* III and cloned into pETduet-1
pDT2570	*rssB*	pETduet-1	Amplified *rssB* using Nde_rssB and rssB_stop_xho, digested with *Nde* I and *Xho* I and cloned into pETduet-1
pDT2575	*rssB*$_N$	pETduet-1	Quick change mutagenesis using pDT2570 and primers RssB_N1 and RssB_N2
pDT2580	*rssB*$_C$	pETduet-1	Amplified *rssB* using Nde_rssBc and rssB_stop_xho, digested with *Nde* I and *Xho* I and cloned into pETduet-1
pDT2324	*iraM & rssB*	pETduet-1	Amplified *rssB* using Nde_rssB and rssB_stop_xho, digested with *Nde* I and *Xho* I and cloned into pDT2467
pDT2574	*iraM & rssB*$_N$	pETduet-1	Quick change mutagenesis using pDT2324 and primers RssB_N1 and RssB_N2
pDT2583	*iraM & rssB*$_C$	pETduet-1	Amplified *rssB*$_C$ using Nde_rssBc and rssB_stop_xho, digested with *Nde* I and *Xho* I and cloned into pDT2467
pDT1783	*iraP*	pET10C	Amplified *iraP* using 5iraP_10C and 3iraP_10C, digested with *Nde* I and *Not* I and cloned into pET10C
pDT2146	*iraD*	pET10C	Amplified *iraD* using 5Nde_iraD and 3iraD_not, digested with *Nde* I and *Not* I and cloned into pET10C
pDT2493	*rssB*$_N$*(D58K)*	pHUE	Quick change mutagenesis using pDT2134 and primers D58K_1 and D58K_2
pDT3124	*iraP & rssB*	pETduet-1	Amplified *iraP* using bam_iraP and iraP_not, digested with *Bam* HI and *Not* I and cloned into pDT2570
pDT3190	*iraP & rssB(D58A)*	pETduet-1	Site directed mutagenesis using pDT3124 and primers D58A_1 and D58A_2
pDT3191	*iraP & rssB(D58P)*	pETduet-1	Site directed mutagenesis using pDT3124 and primers D58P_1 and D58P_2
