# Supplementary figures and images for: Anti-adaptors use distinct modes of binding to inhibit the RssB-dependent turnover of RpoS (σS) by ClpXP
Source: Front Mol Biosci. 2015 Apr 23;2:15. doi: 10.3389/fmolb.2015.00015 (PMC4428439; doi:10.3389/fmolb.2015.00015)

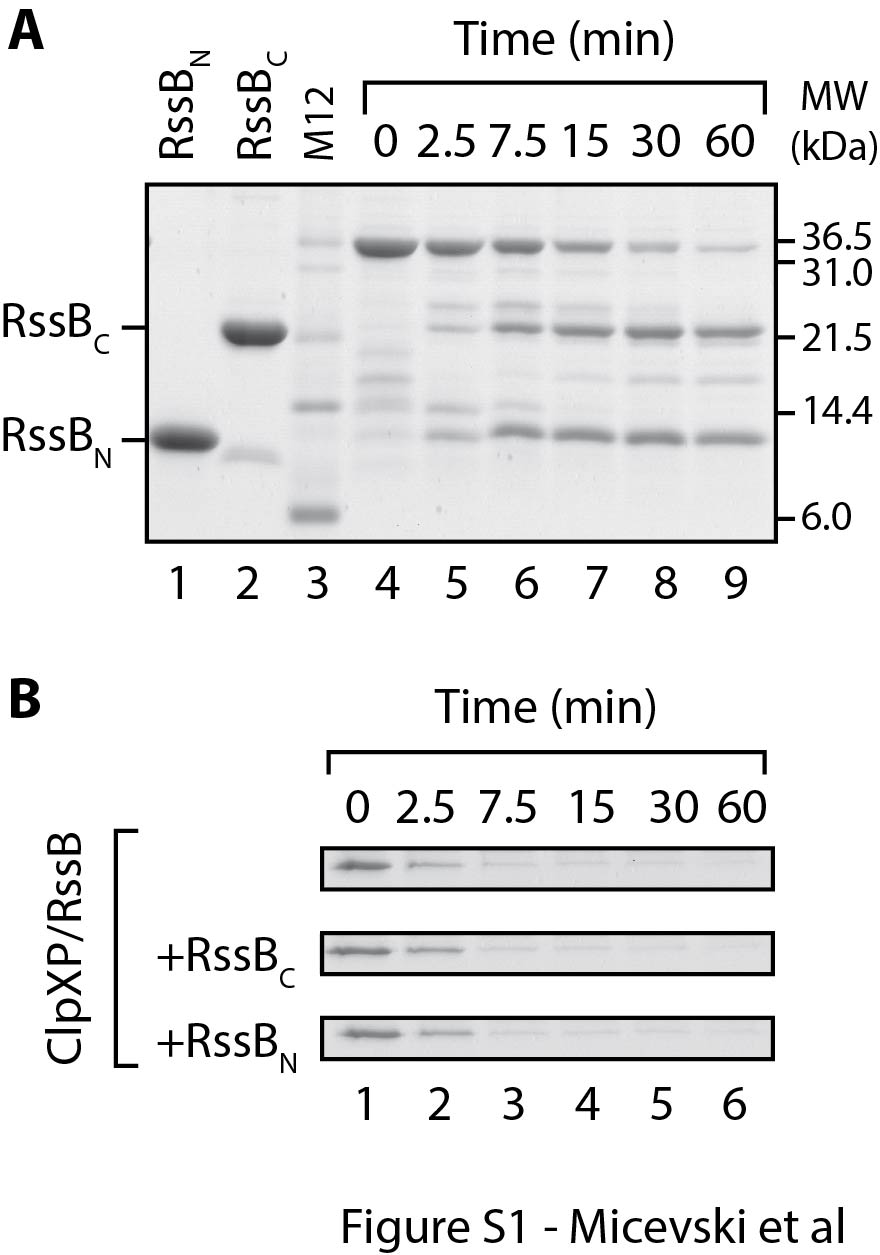

Supplement: Supplementary file 2 [file Image1.JPEG]

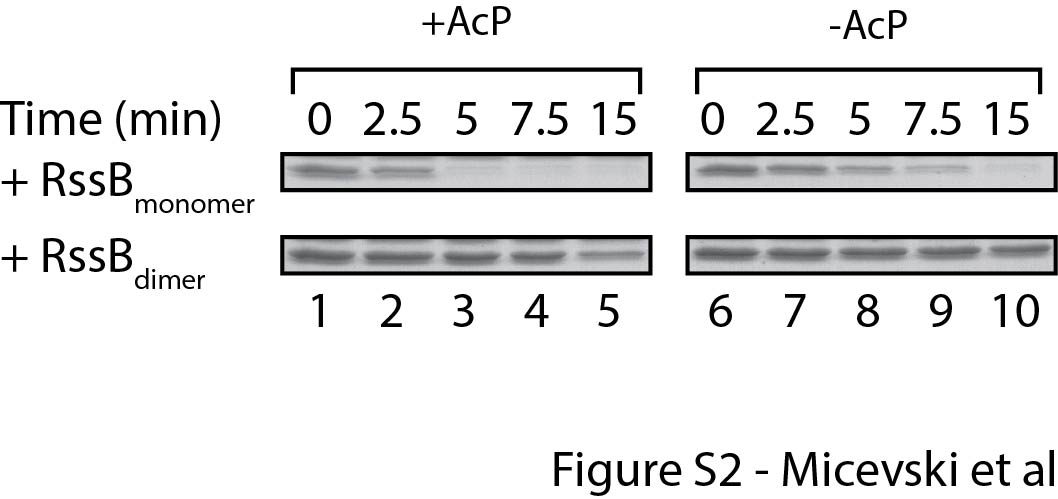

Supplement: Supplementary file 3 [file Image2.JPEG]

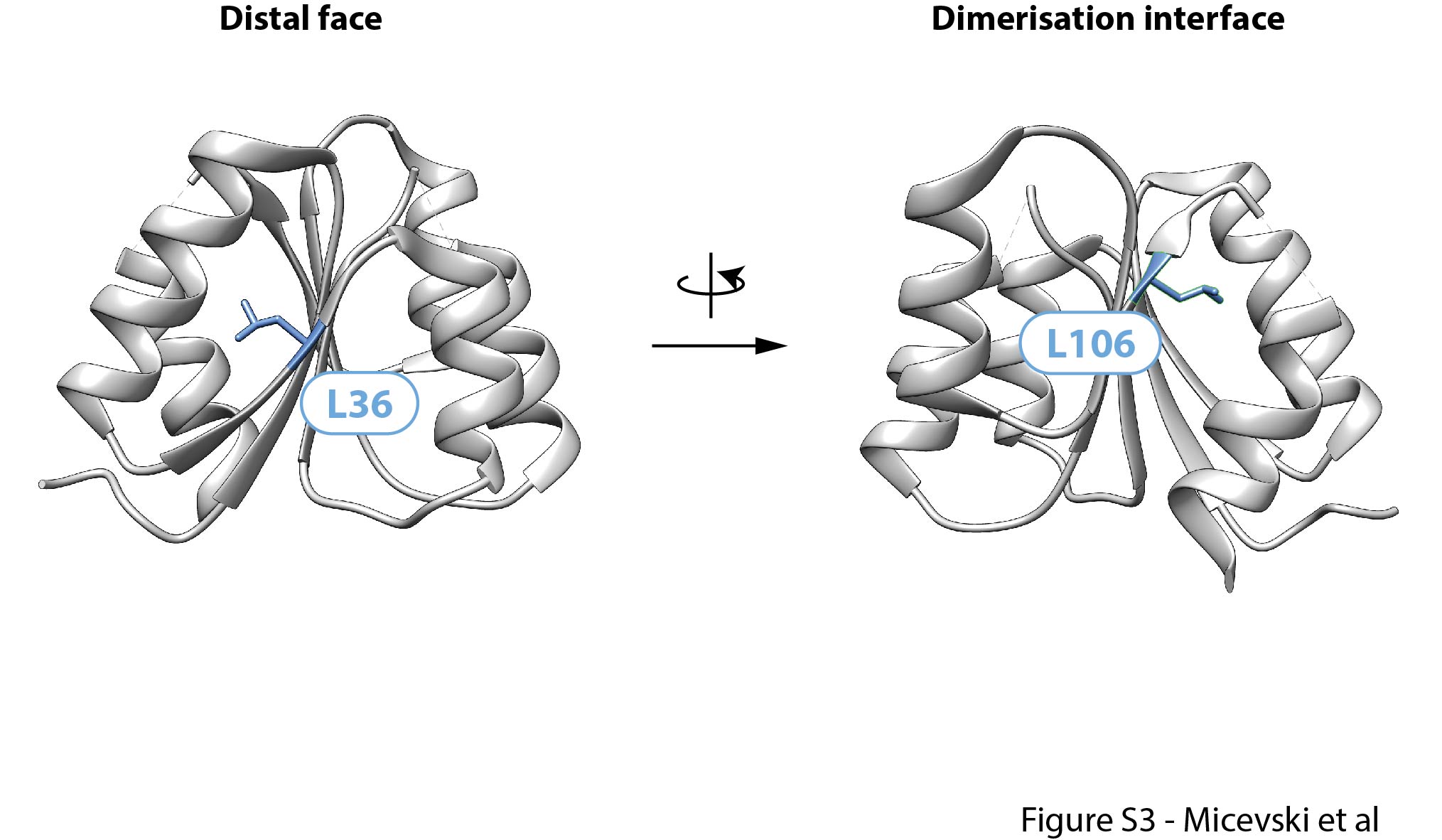

Supplement: Supplementary file 4 [file Image3.JPEG]

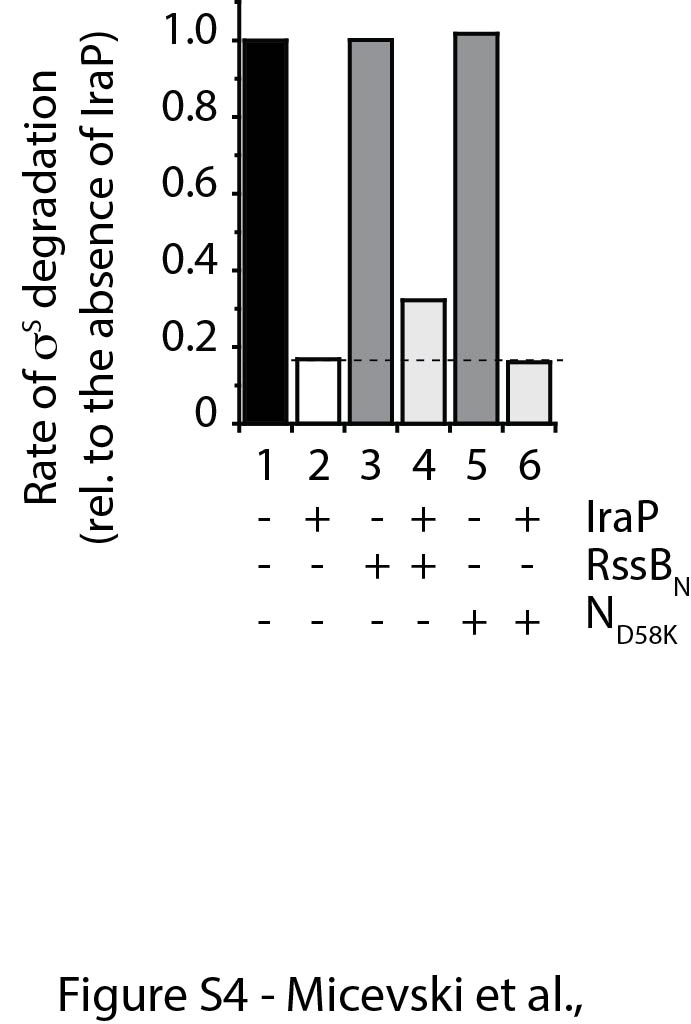

Supplement: Supplementary file 5 [file Image4.JPEG]

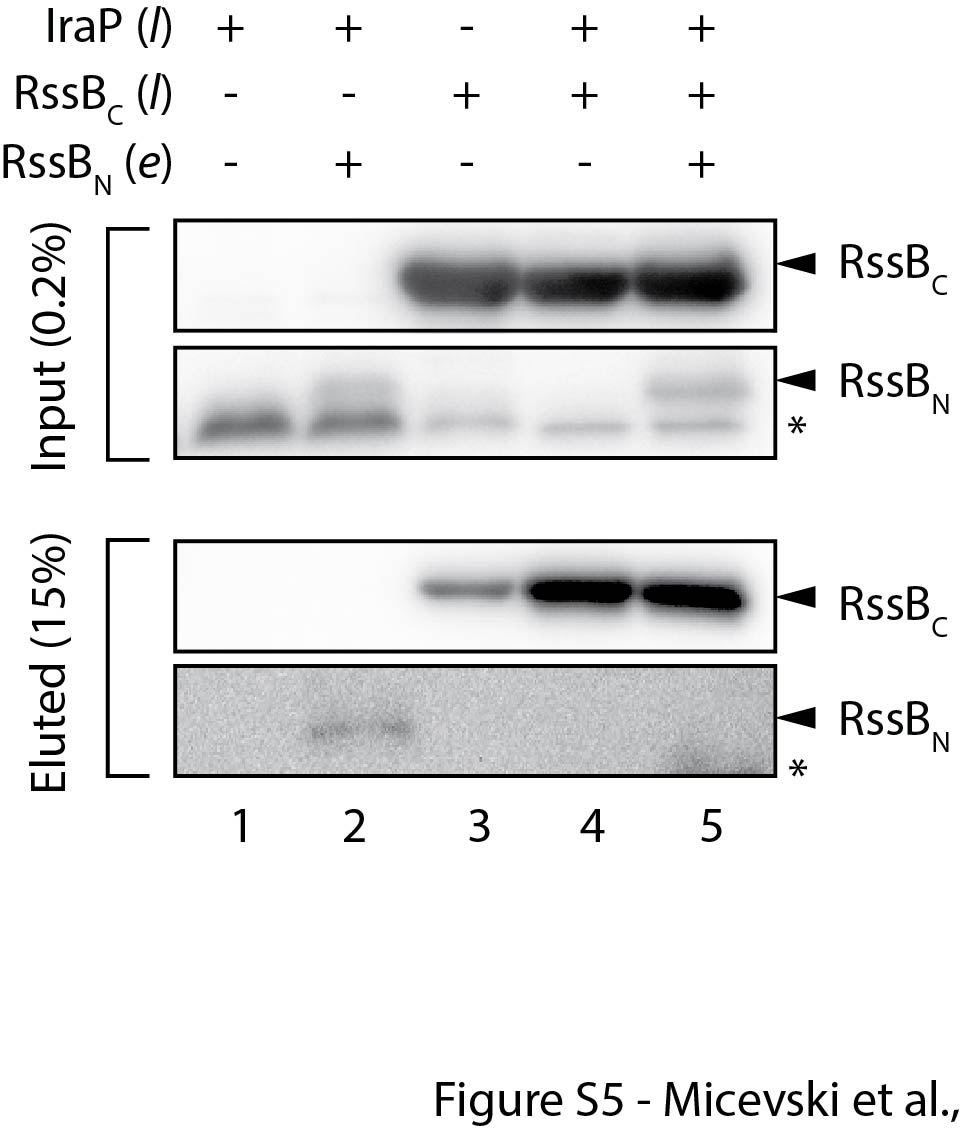

Supplement: Supplementary file 6 [file Image5.JPEG]

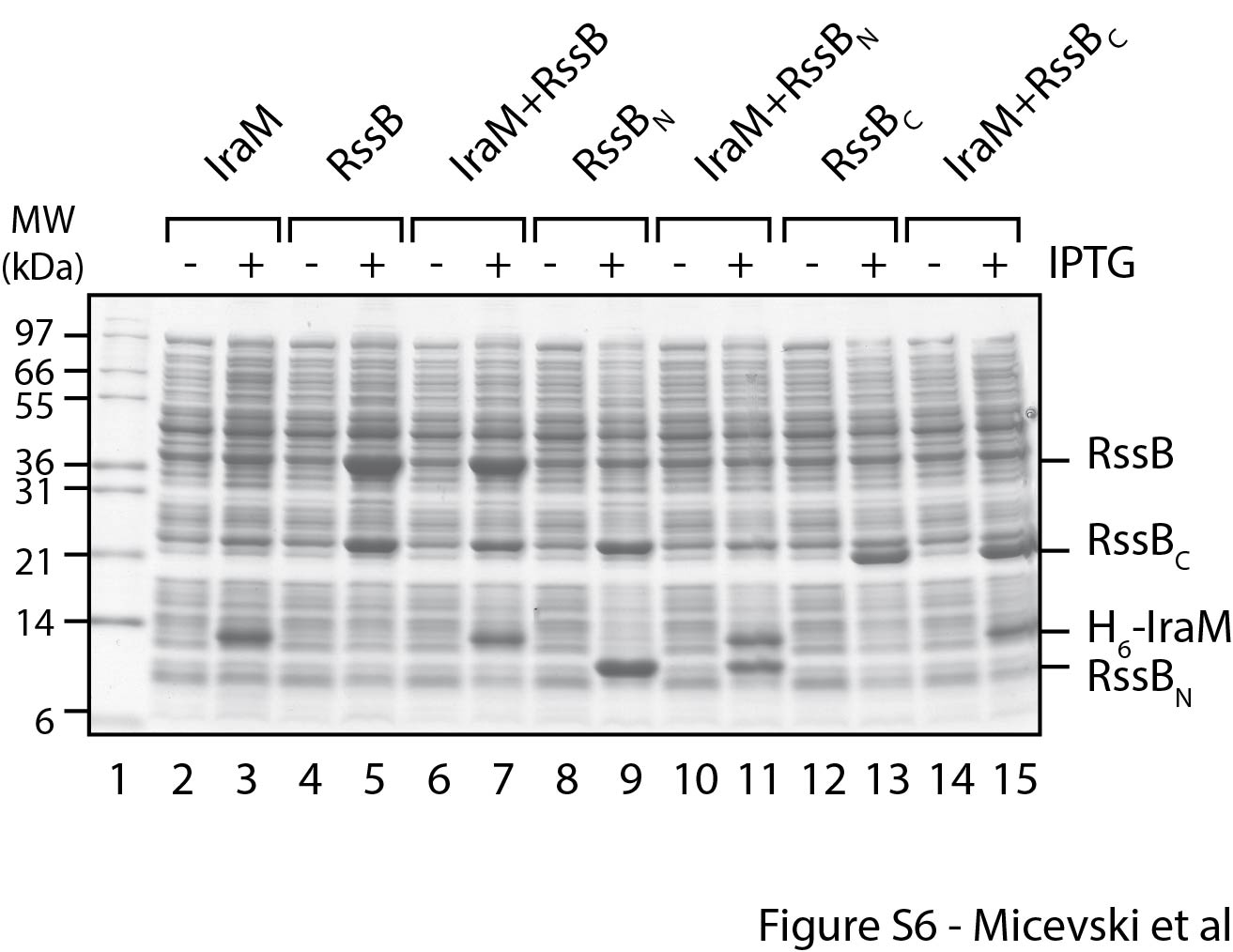

Supplement: Supplementary file 7 [file Image6.JPEG]

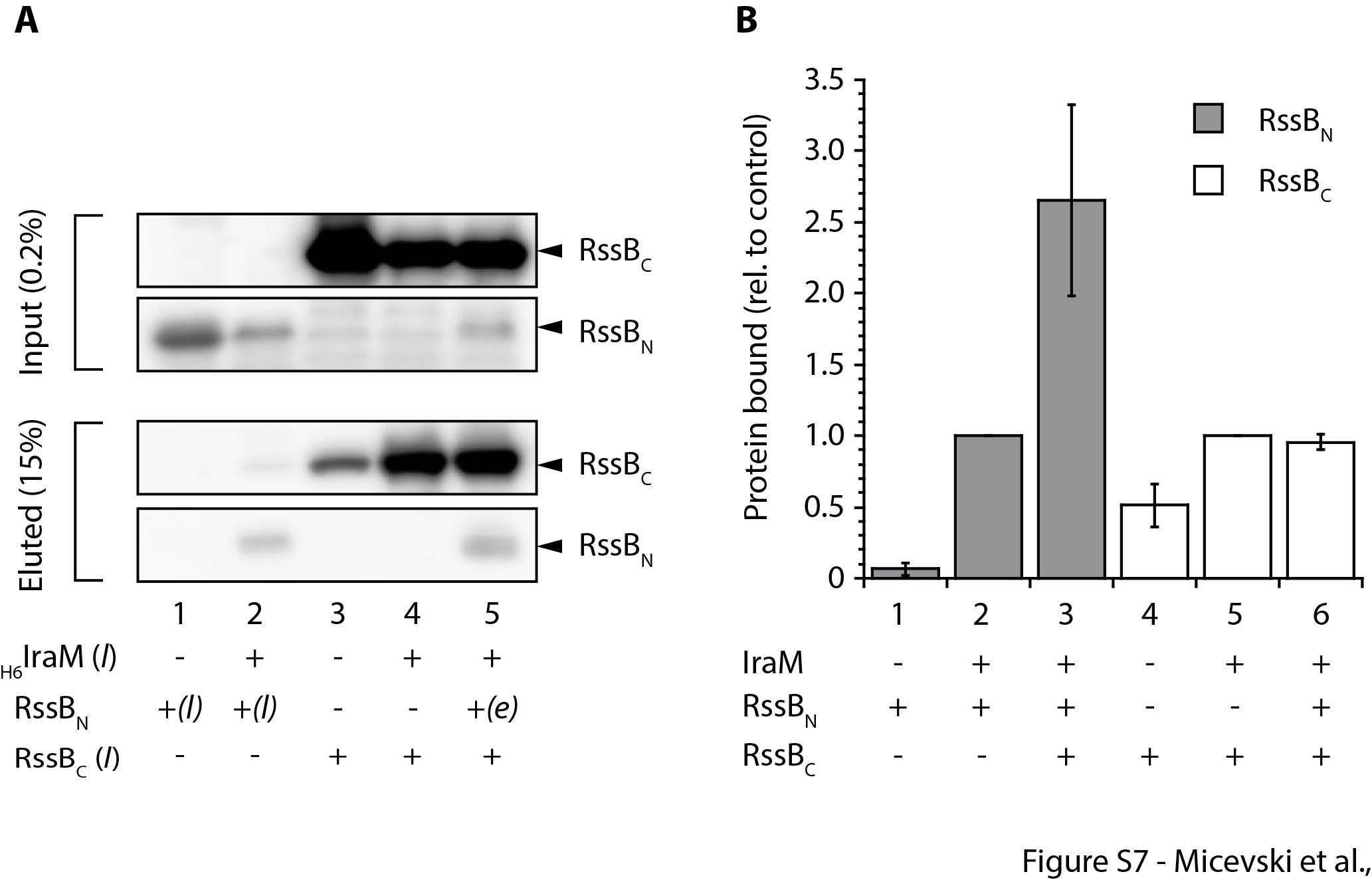

Supplement: Supplementary file 8 [file Image7.JPEG]
